# Supplementary material for: Serine phosphorylation regulates the P-type potassium pump KdpFABC
Source: eLife. 2020 Sep 21;9:e55480. doi: 10.7554/eLife.55480 (PMC7535926; doi:10.7554/eLife.55480)
Supplement: Transparent reporting form [file elife-55480-transrepform.docx]

***eLife’s* transparent reporting form**

We encourage authors to provide detailed information *within their submission* to facilitate the interpretation and replication of experiments. Authors can upload supporting documentation to indicate the use of appropriate reporting guidelines for health-related research (see [EQUATOR Network](http://www.equator-network.org/%20)), life science research (see the [BioSharing Information Resource](https://biosharing.org/)), or the [ARRIVE guidelines](http://www.plosbiology.org/article/info:doi/10.1371/journal.pbio.1000412) for reporting work involving animal research. Where applicable, authors should refer to any relevant reporting standards documents in this form.

If you have any questions, please consult our Journal Policies and/or contact us: [editorial@elifesciences.org](mailto:editorial@elifesciences.org).

**Sample-size estimation**

- You should state whether an appropriate sample size was computed when the study was being designed
- You should state the statistical method of sample size computation and any required assumptions
- If no explicit power analysis was used, you should describe how you decided what sample (replicate) size (number) to use

Please outline where this information can be found within the submission (e.g., sections or figure legends), or explain why this information doesn’t apply to your submission:

The data in Fig. 3 represents a single protein expression for each mutant to survey the effects of alanine substitutions.

The effects of bacterial growth conditions on serine phosphorylation were observed in numerous independent experiments. The data shown in Fig. 4 represents results from five independent expressions.

Measurements of protein activity were measured from multiple independent purifications to ensure the reproducibility of the effects of serine phosphorylation. Data in Fig. 5 include results from an individual experiment (panels A, B, D) as well as data aggregated from 15 such experiments in panel C utilizing purified protein from 3-4 independent cell expressions. Data presented in Fig. 6 were derived from a single preparation with individual data points repeated in triplicate, though analogous results were obtained from at least one independent sample preparation. Data in the Figure Supplements are from individual experiments, but representative of other independent preparations from these same protein constructs.

**Replicates**

- You should report how often each experiment was performed
- You should include a definition of biological versus technical replication
- The data obtained should be provided and sufficient information should be provided to indicate the number of independent biological and/or technical replicates
- If you encountered any outliers, you should describe how these were handled
- Criteria for exclusion/inclusion of data should be clearly stated
- High-throughput sequence data should be uploaded before submission, with a private link for reviewers provided (these are available from both GEO and ArrayExpress)

Please outline where this information can be found within the submission (e.g., sections or figure legends), or explain why this information doesn’t apply to your submission:

With the exception of Fig. 3, activity data were collected in triplicate. Fig. 4 represents data from 5 independent cell expressions. Data in Fig. 6 and in the Figure Supplements were derived from individual protein samples (in triplicate), though they are representative of data obtained from multiple protein preparations from these constructs. Similarly, Fig. 5 includes representative data from individual experiments (performed in triplicate); in addition data has been aggregated in panel C from 15 such experiments that included protein purified from 3-4 independent cell expression for each protein construct.

This information is stated in the respective sub-sections in the Methods section of the manuscript as well as in individual figure legends.

**Statistical reporting**

- Statistical analysis methods should be described and justified
- Raw data should be presented in figures whenever informative to do so (typically when N per group is less than 10)
- For each experiment, you should identify the statistical tests used, exact values of N, definitions of center, methods of multiple test correction, and dispersion and precision measures (e.g., mean, median, SD, SEM, confidence intervals; and, for the major substantive results, a measure of effect size (e.g., Pearson's r, Cohen's d)
- Report exact p-values wherever possible alongside the summary statistics and 95% confidence intervals. These should be reported for all key questions and not only when the p-value is less than 0.05.

Please outline where this information can be found within the submission (e.g., sections or figure legends), or explain why this information doesn’t apply to your submission:

The number of replicates for each experiment is described both in the Methods section and in the figure legends. The program used for statistical analysis (Prism8) is specified both in the Methods section and in the relevant figure legend.

(For large datasets, or papers with a very large number of statistical tests, you may upload a single table file with tests, Ns, etc., with reference to sections in the manuscript.)

**Group allocation**

- Indicate how samples were allocated into experimental groups (in the case of clinical studies, please specify allocation to treatment method); if randomization was used, please also state if restricted randomization was applied
- Indicate if masking was used during group allocation, data collection and/or data analysis

Please outline where this information can be found within the submission (e.g., sections or figure legends), or explain why this information doesn’t apply to your submission:

**Additional data files (“source data”)**

- We encourage you to upload relevant additional data files, such as numerical data that are represented as a graph in a figure, or as a summary table
- Where provided, these should be in the most useful format, and they can be uploaded as “Source data” files linked to a main figure or table
- Include model definition files including the full list of parameters used
- Include code used for data analysis (e.g., R, MatLab)
- Avoid stating that data files are “available upon request”

Please indicate the figures or tables for which source data files have been provided:
